# Description of an advance care planning intervention in nursing homes: outcomes of the process evaluation

**DOI:** 10.1186/s12877-018-0713-7

**Published:** 2018-01-25

**Authors:** Irene Aasmul, Bettina S. Husebo, Elisabeth Flo

**Affiliations:** 1 Centre for Elderly and Nursing Home Medicine, Department of Global Public Health and Primary Care, University of Bergen, P.O. Box: 7804, Bergen, Norway; 2 Department of Nursing Home Medicine, Municipality of Bergen, Bergen, Norway; 3 Department of Clinical Psychology, University of Bergen, Bergen, Norway

**Keywords:** Advance care planning, Education, Implementation, Nursing home, Staff training, Train-the-trainer

## Abstract

**Background:**

Advance Care Planning (ACP) is the repeated communication and decision-making process between the patient, family, and healthcare professionals. This study describes an ACP intervention in nursing homes and evaluates the outcomes of the implementation process.

**Methods:**

The ACP intervention was part of a 4-month complex, cluster randomized controlled trial (COSMOS). 37 Norwegian nursing homes with 72 units (1 cluster = 1 unit) and 765 patients were invited to participate and eligible units were randomised to the intervention group or control. Nursing home staff in the intervention group was offered a standardized education programme to learn early and repeated communication with patients and families and to implement ACP in their units. We used a train-the-trainer approach to educate staff in the units, supported by regular telephone calls and a midway seminar after two months. Individual patient logs consisting of different communication deliverables were used to evaluate the implementation process. Supported by Qualitative Content Analyses, we identified facilitators and barriers of the ACP implementation based on feedback during midway seminars and individual patient logs.

**Results:**

The ACP intervention was conducted in 36 NH units (*n* = 297); 105 healthcare providers participated at the education seminar prior to the study, and 3–4 employees from each unit participated in the midway seminar. NH staff reported the educational material relevant for the implementation strategy. The patient logs showed that ACP was successfully implemented in 62% (*n* = 183) of the patients using our predefined implementation criteria. The staff emphasized the clear communication of the relevance of ACP addressed to leaders and staff as important facilitators, along with the clearly defined routines, roles and responsibilities. Identified barriers included lack of competence, perceived lack of time, and conflicting culture and staff opinions.

**Conclusion:**

Monthly communication with the family was the most frequently conducted communication, and the predefined criteria of successfully implemented ACP were largely achieved. Nursing home routines and engagement of leaders and staff were crucial facilitators, whereas lack of time and competence reduced the implementation success.

**Trial registration:**

The COSMOS-trial was registered in the ClinicalTrials.gov (NCT02238652) July 7th, 2014

**Electronic supplementary material:**

The online version of this article (10.1186/s12877-018-0713-7) contains supplementary material, which is available to authorized users.

## Background

Death is inevitable. However, medical progress has postponed and institutionalized the last period of life. With considerable variations between different countries, figures demonstrate that most deaths in Europe occur in nursing homes (NH) or hospitals [1–3]. In general, NH patients are characterized by multimorbidity and polypharmacy; most of them have dementia [4–7]. When dying is imminent, these individuals are no longer able to participate actively in medical and ethical decision-making. Consequently, nursing home patients with and without dementia depend on others to make qualified choices at the end-of-life [6].

Advance Care Planning (ACP) is a repeated communication process between the patient, family, and healthcare professionals to evaluate the individual preferences, values and goals, and potential concerns about treatment and care of the patient [8–10]. This procedure is approached by shared decision-making, including NH patients with dementia and their family to take part in in this process [11, 12]. The ACP concept is based on the patient’s basic human right to be informed about her disease and treatment options, in order to make informed decisions [13, 14]. In people with dementia, the conversation should preferably start while the patient is still capable of active participation. If a patient has no ability to provide informed consent, it is a key premise that the family are empowered to make informed presumed decisions following the question “What would my loved one have wished in this situation?” [15].

Early communication increases the opportunity to respect the patient’s and family’s needs and preferences in the light of their spiritual and cultural background [16]. The number of studies investigating the implementation and efficacy of ACP is limited [17]. This may in part be due to its complex nature, along with variations in terms of study design, setting, and outcome measures. The goals of ACP may also vary between different countries and depend on spiritual and cultural implications, and values and legal systems [9]. Meanwhile, only a few countries have developed and implemented an official standard for the ACP process [18], leading to a more coincidental communication between staff and family [9, 15].

In a recent systematic review, we identified 16 ACP studies conducted in NHs [9]. A Hong Kong-based study using the “Let me Talk” programme found that patients in the intervention group (*n* = 59) communicated their treatment preferences more frequently compared to controls [17]. However, involving the participants’ family in the ACP conferences was challenging because the patients suggested that family members were too busy to spare the necessary time to attend [17]. Another study by Cornally et al. (2015) utilised the “Let Me Decide” programme in three NHs in Ireland, leading to enhanced communication and prevented last-minute decisions. The study however, reported barriers associated with lack of physician involvement and difficulties using the required screening instrument to assess the patients’ capacity to consent [19]. Our review also highlighted the lack of staff competence as a key challenge, rendering education a prerequisite for proper ACP implementation [9].

In a complex intervention like the ACP, it is highly necessary to describe and assess the evidence-based implementation strategy [20, 21]. The strategy of ACP is just as important to describe as its content, yet few studies have investigated the strategy and definition of implementation [9, 22]. The aim of this study was to describe the content of ACP in the COSMOS study, as well as the evaluation of the implementation process of the intervention in Norwegian NHs, using the following research questions:

1. How did the NH staff accept the ACP intervention and implementation strategy?
2. To what degree was the ACP intervention implemented successfully?
3. What were the barriers to and facilitators of implementing ACP in the NH?

## Method

The ACP intervention described in this study was a part of the COSMOS trial, a 4-month complex randomised controlled trial executed from August 2014 to December 2015. The COSMOS acronym refers to the trial components: *CO*mmunication (in the form of Advance Care Planning), *S*ystematic assessment and treatment of pain, *M*edication review, *O*rganization of activities, and *S*afety [23]. We invited eight Norwegian municipalities from three counties, including 37 NH with 72 NH units (1 unit = 1 cluster) and 765 NH patients to participate. Eligible NH units were randomised to the intervention or control group. In the current study, we focus on the intervention group, by describing the content, implementation strategies of the ACP intervention, and the outcomes of the evaluation process. To achieve a representative sample, both somatic and specialized dementia long-term units from rural, urban, rich and poor municipalities were invited. Patients who were 65 years or older and had a minimum stay of two weeks before assessment were eligible participants. Patients with life expectancy ≤ 6 months or with schizophrenia were excluded. For detailed information on study design and sample size analyses, please see the published COSMOS protocol [17]. NH managers, registered and licensed practical nurses, and physicians related to the intervention group were invited to participate in a two-day education seminar, which offered a standardized education programme about ACP with patients and families. Nurses attending the education seminar were named COSMOS ambassadors.

### Content of the advance care planning intervention

The content of the ACP intervention was based on literature reviews, clinical experience, and national and international collaboration [9, 22, 24]. The content was guided by the aim of achieving rapport and trust between NH staff, patient and families, to allow a necessary clarification of the patient’s values and needs, and to achieve quality of life and quality of dying. We designed the ACP intervention so that most patients, including those with dementia, would benefit from being included in ACP discussions [25]. The ACP content included an open and clear communication about the patient’s medical conditions, treatment choices, possible disease trajectories, and potential future medical decisions (Table 1). To facilitate initiation and ensure that the staff also asked the “difficult questions”, we provided seven questions to cover important themes to be introduced in the conversation (Table 2). Due to the high prevalence of patients with advanced dementia in Norwegian NHs [6], these questions were created with family members in mind. It focused on the importance of achieving knowledge and to bring out the patient and the families’ preferences not only for specific types of treatment but also for a focus on life values [12]. Timing and sensitivity to the patient and family’s current situation and understanding of the patient’s health status was therefore emphasized [26]. The intervention stressed that it was beneficial to create space for patients and families to discuss these issues as early as possible, instead of postponing them until a crisis required decisions to be made. Importantly, we did not recommend that decisions should be finalized at an early stage, when they do not yet appear relevant to the patient or family (e.g., use of antibiotics, or use of morphine at the end-of-life).

**Table 1:** The main themes of the education programme

• Understanding the definition and perspectives of Advance Care Planning, and potential consequences of not providing Advance Care Planning • Topics that the Advance Care Planning should cover and how to identify the patients’ needs • Potential challenges related to nursing home patients and their family (e.g. dementia, loss of capacity to consent) • How to involve families, and initiate the communication process • Basic considerations to ensure good communication (e.g. open-ended questions versus closed-ended, attentive listening, providing both written and verbal information) • The necessity of organizing formal meetings and not only informal (coincidental) communication • Practical considerations (e.g. the use of a meeting room to ensure suitable facilities and good atmosphere) • Documentation of communication to ensure adherence in practice

**Table 2:** Seven key questions and themes in Advance Care Planning^a^

1.	How involved have you been in the patient’s treatment, care and decision-making as family, and how much would you like to be included?
2.	What have both of you (patient and family) understood about the situation and the disease?
3.	What kind of additional information do both of you (patient and family) need so as to better understand the situation?
4.	What should we know about the patient’s life and values to ensure the best care? What matters and what makes life in general meaningful?
5.	What goals, ideas and expectations do you both (patient and family) have for the nursing home stay?
6.	Does the patient struggle with unfinished business*?*
7.	Have both of you (patient and family) previously discussed end-of-life treatment e.g. hospitalization in case of acute illness?

^a^The questions were listed in the ACP flash card, available as an online Additional file 2

**Fig. 1:**
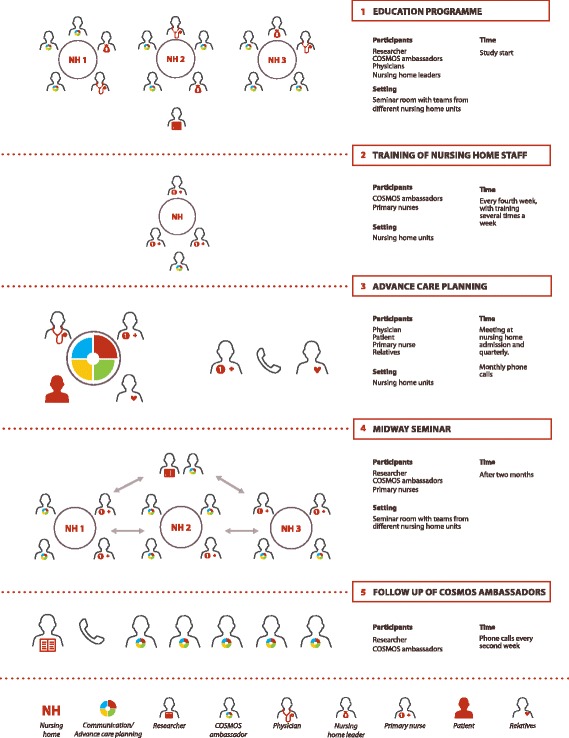
Implementation of Advance Care Planning. Legend: Overview of the COSMOS ACP implementation process in the different steps. Step 1: Gathering the intervention group to the education seminar. Step 2: COSMOS ambassadors training the staff back in the NH unit. Step 3: ACP, involving patient, family, nurses and physicians by meetings, and regular phone calls. Step 4: Gathering COSMOS ambassadors and primary nurses to a midway seminar. Step 5: Researchers’ follow-up of COSMOS ambassadors every second week

*Frequency of ACP communication* was clearly defined in the COSMOS ACP intervention (Fig. 1, step 3): (i) a meeting with the physician and primary nurse was offered within 2–3 weeks after admission and subsequently repeated quarterly, (ii) telephone contact was maintained with the family on a monthly basis (could be replaced by talks at the unit). Meetings and calls should be conducted when the patient had good periods, and if the patient could only participate in parts of the meetings, the conversation should be held with the family [27, 28]. The frequency of contact was operationalized based on the frailty of the population, with a potentially rapid deterioration in health and cognitive functioning.

*Roles and responsibilities* were clearly defined. The primary nurses were responsible for organizing meetings and maintaining the frequency of contact, which is considered a realistic task, provided proper training (Fig. 1, step 3) [22]. We emphasized that it was optimal to provide verbal and written information in the form of a meeting agenda when organizing the meetings. Based on previous research, we emphasized the value of having the physician, preferably with an established patient relationship, attend the quarterly meetings [29–31].

*Documentation* of the ACP communication was a vital element of the intervention. It was stressed that information regarding the patient’s and family’s preferences or thoughts on medical decisions should be documented and readily available for the staff on duty. Since different NHs used different record systems and routines, it was stressed that the COSMOS ambassadors should discuss with their colleagues and manager, how their documentation could be improved so that vital information was available in case of an emergency.

## Implementation of advance care planning

### Education programme

The content of the two-day education seminar was based on previous studies, systematic reviews, and clinical experience [9, 22, 24]. At least two nurses from each NH unit, with hands-on experience with NH patients were required to attend the education. These participants were titled COSMOS-ambassadors (Fig. 1, step 1). The seminar was led by two of the researchers (BSH and EF). It included lectures, skills training, role-play, and rehearsed use of the implementation material (see step 1 in Fig. 1) [23]. The role-play aimed to create realistic situations and experiences on the use of open-ended questions to clarify the individuals’ understanding and needs. The programme stressed that details in the formulated questions must be adapted to the patient’s cognitive function [13, 14]. It was also stressed as essential that questioners must be attentive to the patient’s responses, e.g. non-verbal reactions, frustration and uncertainty as a response to the use of open-ended questions especially if discussing different choices [15–17]. An important principle provided in the education was to include both patient and family in the shared conversations with the healthcare providers, even if the patient had cognitive impairment. Challenges and advantages of this principle were discussed [26]. However, an important part of the ACP education explained the need for flexibility and adaption in the communication process to ensure that both family and patient had the opportunity to partake in the decision making.

### Training of NH staff

A train-the-trainer focus [32] involving the whole nursing staff in the unit was employed to ensure that the ACP implementation was sustainable [33]. After the two-day seminar, the COSMOS ambassadors were responsible for teaching their colleagues in the unit about the ACP process (Fig. 1, step 2). Based on barriers identified in our review [9], the implementation strategy did not depend on one single person, i.e., the ambassador, but also on the primary nurses and NH managers. Although time-consuming, it is crucial to educate staff in order to facilitate change and development in large organizations [34]. The ambassadors were encouraged to find an optimal setting, according to their local routine, in which to train colleagues (see Fig. 1 step 2). The researchers advised the ambassadors to talk during lunch and/or report (10–20 min.), several times per week to enable optimal coverage [35]. Since ACP was one of four COSMOS components, the ambassadors were advised to keep a focus on ACP every fourth week. However, staff were encouraged to organize an ACP meeting regardless of the week (Fig. 1, step 3).

### Training material

During the two-day education seminars, the ambassadors received training material, including guidelines, educational binders, and flash cards (see Table 3). The wording in all of the training material was adapted to both registered and licensed practical nurses, aiming to appeal to all caregivers responsible for the patient.

**Table 3:** Advance Care Planning material used to train the NH staff in the unit

What	Content
Guidelines	A booklet (two per unit) was provided to describe the content of ACP, with evidence-based facts and referenced literature.
Educational Binder	Abridged power point slides from the two-day education seminar, collected in an educational binder, used for teaching colleagues.
Flash cards	Cards which fitted in the staffs’ pocket, reminding staff of the main focus of ACP; what it is, who should participate and how often communication should be initiated. Cards also included examples of suitable questions and themes (Table 2) to be discussed in meetings.

### Midway seminar

All intervention units were invited to a midway seminar after 2 months (Fig. 1, step 4), led by the research team. A brief lecture to review the ACP intervention was provided. Further, the COSMOS ambassadors presented their successes and challenges in a plenary session so that experiences, support and ideas were discussed and shared between the participating units. We used the metaphor of a traffic light to identify and organize areas of success and challenges; each NH-unit noted three successes in the implementation process “green light”, two challenging elements “yellow light”, and one element not completed at all “red light” (See traffic light illustration as online Additional file 1).

### Follow-up of COSMOS ambassadors

Researchers were in contact with the NH units during the intervention period by means of regular telephone contact every second week to support the implementation (Fig. 1, step 5). In addition, there was a telephone/email hotline (Monday to Friday 08:00–16:00).

### Assessments and implementation outcomes

The NH managers filled in a questionnaire on the NH’s prior participation in research projects or quality improvement initiatives. Staff demographics and characteristics were collected for all staff involved in data collection at the NH units. Patient demographics and characteristics were recorded from the patient records. Data were collected at baseline, and at month 4.

The patients’ cognitive status was assessed by researchers using the Mini Mental State Examination (MMSE). This test provides a sum score ranging from 0 to 30 and can be used for case detection using cut-off scores, i.e. 26–30 normal cognition, 21–25 for mild dementia, 11–20 for moderate, and 0–10 for severe dementia [36, 37]. MMSE has high test-retest reliability, internal consistency and inter-rater reliability [36–39].

The acceptance of the ACP intervention was assessed by investigating whether participants attended the education programme, midway seminar and how they used the individual patient log. Further information was collected during the regular telephone call every second week. The primary nurses were responsible for documenting the implementation by filling in an individual log for each patient every fourth week. Questions in the logs are listed in Table 5. These logs were used to evaluate the outcome of the implementation process. The ACP intervention was defined as successfully implemented if the tasks in the log corresponding to questions 1 or 2 *and* 3 or 4 were completed during the 4-month trial period. The logs assessed the key ACP intervention deliverables with the following questions (Yes – No – Not applicable):

1. Have the patient and family been invited to a conversation with the physician?
2. Have the patient and family had a shared conversation with the primary nurse?
3. Have there been monthly phone calls to the family?
4. Have you had contact with the family the last month?
5. Is the communication documented?

Additionally, there was a free text area in the logs to write comments. Together with the assessment of facilitators and barriers from the logs and the midway seminar, this provided insight about how this study contributed to implement the ACP intervention [35].

### Analyses

Statistical analyses were performed using IBM SPSS Version 23. The demographics for NH staff, patients, and clinical characteristics were summarized using means and standard deviation (SD) or frequencies and percentages.

The implementation outcomes, including entries to each intervention deliverable in the log were summarized as frequencies and percentages for the whole 4-month period and for each time point (months 1, 2, 3 and 4).

The free text feedback from the logs and discussions in the midway seminar were transcribed. Categories and sub categories were identified, based on Qualitative Content Analysis in accordance to the research question: identifying facilitators and barriers [40]. Three researchers (IA, EF, and BH) read and analysed the data individually and registered the main topics that emerged [41]. Topics were identified by attending to prominent and recurring themes. Identified categories and sub categories of facilitators and barriers were then cross-compared and discussed until consensus was achieved between the researchers [41], this process was intended to achieve trustworthiness with agreement between the researchers [42]. Meaningful quotations were extracted from conversations with the nurses during the midway seminar.

## Results

One municipality with four NHs declined to participate. In addition, one unit withdrew from the study, leaving 67 units from 33 NHs with 723 patients to be randomized. We subsequently excluded 178 patients due to: lack of consent (151), age < 65 years (15), moved before study start (6), terminal condition at study start (2), death before study start (1), schizophrenia (1), withdrawal of consent (1), and unknown reason (1). Accordingly, 545 patients from 67 units were included in the main study. In this paper, we investigated those receiving the intervention and implementation strategy, i.e., the intervention group, including 36 NH units, with 297 patients. All results reported below, relate to the intervention group.

### Nursing home units and staff characteristics

One unit had previously participated in a research project on communication and end-of-life care, while three units had carried out local initiatives. The staff coverage was 3.2 patients each at daytime (range 1.6 to 4.0), 4.7 (2.3–6.0) in the evening, and 13.0 (4.0–30.3) at night-time. The staff (*n* = 67) had worked an average of 18 years (SD = 10.8) in the healthcare sector, and 9 years (SD = 7.3) in the current NH unit. Close to 80% (*n* = 52) were registered nurses of whom 22% (*n* = 15) had additional education. More than 20% (*n* = 14) did not have Norwegian as their first language, originating from Europe (*n* = 8, 12%) and Southeast Asia (*n* = 6, 9%).

### Patients’ characteristics

The included patients (*n* = 297) had a mean age of 86.5 (SD = 7.7) years, 73% (*n* = 216) were females. As shown in Table 4 the mean MMSE score was 10.4 (SD = 7.6) and 141 (47%) patients had severe dementia [37]. During the 4-month intervention period, 33 (11%) patients died in the intervention group, 14 (5%) moved, and 13 (4%) patients were hospitalized.

**Table 4:** Patients characteristics

	Patients (*n* = 297)
Age, mean (SD)	86.5 (7.7)
Females, N (%)	216 (73)
Cognition, N (%)	
MMSE	
Normal	9 (3)
Mild dementia	21 (7)
Moderate dementia	107 (36)
Severe dementia	141 (47)

MMSE: Mini Mental Status Examination

The sums of percentages of the MMSE score are not 100, due to missing values

### The acceptance of the advance care planning intervention

Due to different starting points of the study for the nursing homes, four 2-day education seminars were completed with 105 persons attending, of which 74 were staff with daily patient contact. All participating units sent the required two nurses to the education seminar (44 registered and 9 licensed practical nurses); 7 of the 21 invited physicians attended, 24 NH managers participated, while 21 participants had other or unknown occupations. Most COSMOS ambassadors expressed that the ACP flash card was very useful. The feedback on the binders was mixed; some expressed that they were too comprehensive, while others enjoyed the opportunity to learn more about ACP. At the midway seminar, all 36 units participated with 3–4 NH employees from each unit (both COSMOS ambassadors and primary nurses, but no physicians). Participants reported these seminars to be helpful.

### Results of the implementation of advance care planning

All units used the patient logs. 19% (*n* = 56) of the patients had no log entry over four months because most of them (77%) had moved or died during this period. As the total number of patients remaining in the study declined, the total number of patients changed throughout the intervention period. For the first four weeks, the log was filled in for 73% (215 of 294) of the patients, at week 8: 72% (206 of 288), at week 12: 50% (137 of 276) used it, and at week 16: 73% (198 of 271). Successful implementation was achieved in 183 (62%) of the patients by month 4, which means that they had fulfilled the following criteria: patient and family were invited to a meeting with the physician or the primary nurse, and family was contacted monthly by phone or in the unit. Monthly communication with family (*n* = 165, 76%) and documentation of the communication (*n* = 217, 73%) were the two most frequently conducted items (Table 5).

**Table 5:** Response in the patient logs during the intervention period of 4 months; *n* = 297^b^

	Yes		No		Not applicable/ Don’t know	
	N	%	N	%	N	%
Implemented Advance Care Planning^a^	183	62%	58	20%	0	
1. Have the patient and family been invited to a conversation with the physician?	98	33%	135	45%	7	2%
2. Have the patient and family had a shared conversation with the primary nurse?	166	56%	72	24%	2	1%
3. Have there been monthly phone calls to the family?	165	56%	62	21%	12	4%
4. Have you had contact with the family the last month?	226	76%	12	4%	2	1%
5. Has the communication been documented?	217	73%	19	6%	2	1%

^a^ACP was defined as implemented if units had completed questions 1 or 2 and 3 or 4, during the 4-month trial

^b^Due to missing data the number of participants does not add up to 297 per item

### Facilitators for the advance care planning implementation

Based on patient logs and feedback at the midway seminars, we identified two main categories of facilitators: the clearly defined impact on routines, roles and responsibility, as well as the clear communication of the relevance of ACP (Table 6). The staff reported that the intervention’s focus on institutional organization and routines, with clearly defined roles and responsibility, was helpful. For example, the primary nurses were defined as responsible for establishing communication and organizing ACP meetings for “their” patients. This left no room for arguing about “who should have done what” to achieve such meetings. The staff also reported that the specified routines for communication suggested in the ACP education (e.g. asking open-ended questions, attentive listening and using the seven key questions and themes) helped guide them to initiate and maintain contact.

*The questions from the flash cards have helped a lot, to use as introductory questions. (They) made it much easier to address individual wishes concerning end-of-life and preferences for the individual in their daily life.*

**Table 6:** Facilitators of and barriers to implementing ACP in the nursing home unit

Facilitators:
• Clear impact on the organization, routines and responsibilities: - Systematic involvement of nursing home managers - Systematic training of all staff in the unit to clarify new routines - Assigning responsibility to all primary nurses - Routines for dialogue between the physician and nurses (clarifying responsibilities) - Enabling agreement on documentation - Clear schedules for internal training - Clear schedules for conversation with patient and family - Clarified routines for including the patient in relevant discussions - Routines for communications: e.g., telephone and email - List of questions to clarify the needs for the patient and family, including the family’s preferences for involvement - A specified routine for contacting the family without a specific reason - Defined space in staff schedule to discuss ACP as an important topic
• Clear communication of the relevance and need for education regarding ACP: - The education conveyed ACP as important and inspiring - Education showed in what way there was potential for improvement - The training material was understandable and improved the competence on ACP - Flash cards were interesting and easy to use, even when time was limited
Barriers:
• Lack of time: - to teach colleagues in the unit - for the physician to participate at the two-day education seminar, and meetings
• Conflicting opinions and culture: - The patient considered not capable to participate at a shared conversation - Perception of already sufficient contact with family
• Lack of staff competence: - Challenging to engage staff with lower education and understanding of ACP - Difficult to get everyone to read the documentation in the journal - Lack of documentation skills - Lack of Norwegian language skills - Too large quantity of training material for part-time or uneducated staff - High level of sick leave among staff leading to unskilled replacements

The new routines for monthly contact helped the staff to keep families systematically updated, which substantially improved the contact with family members, including those living far away. The relevance and need for education regarding ACP was highlighted in the education seminar and material. The content of ACP was considered relevant for the daily work in the unit, and the education seminars and material were often experienced as spot-on for practical use. This helped the ambassadors to convey “the message” convincingly.

*Talking through these questions provides a high level of assurance and the families thought it was very good to use time to talk about these subjects. It leaves few things unsaid.*

### Barriers to the advance care planning implementation

Lack of time emerged as a prominent barrier, particularly time to train and involve colleagues. Furthermore, because few physicians found the time to take part in the two-day seminar, it was difficult to motivate them to participate in the intervention. Other barriers were: existing culture and staff opinions, that conflicted with the ACP intervention. For example, a common opinion was that patients with dementia should not participate in ACP conversations, while this was promoted in the ACP intervention. Lack of competence among staff also emerged as an important barrier. For example, the ambassadors experienced that untrained staff did not understand the significance of the ACP intervention, and were thus not motivated to read the guidelines and engage in the training. In addition, some staff had poor Norwegian language competence, which affected both their ability to understand the ACP content and to have sensitive conversations. The cultural aspect of not being accustomed to discussing delicate matters like end-of-life care was also prominent.

## Discussion

This study describes the content and implementation strategy of an ACP intervention in Norwegian NHs including patients with and without dementia. Based on the patient logs, 62% of patients and their families fulfilled the predefined criteria of having received ACP. The intervention was well received among the staff and they gave positive feedback on the close follow-up with support from the researchers every second week. The gathering of different NH units at the midway seminar motivated them to keep up the implementation of ACP. We identified facilitators, including the convincing communication of the relevance of ACP to leaders and staff, and the clearly defined routines, roles and responsibility. We also identified relevant barriers that may hinder a proper implementation process, such as lack of time, lack of competence, and conflicting culture and staff opinions. Furthermore, we discovered challenges in engaging NH physicians, as they had less time or interest to participate in the education programme and the conversation with patients and families. These results are of key importance, as they provide information on the implementation process, useful for the practical field.

This process evaluation study achieved a somewhat high implementation rate of 62%, although the result was difficult to compare with other studies due to the different operationalization of ACP. Previous studies have defined ACP as fully implemented if a legally binding document for future medical decisions (e.g., advance directive) has been completed [43, 44]. For example, in the study by McGlade et al. (2017), approximately half the participants (*n* = 290 patients) were reported to have received ACP, when implementation was defined as a completed end-of-life-form [44]. A legal document/directive is more relevant in countries with legislative and cultural pressures [9]. In Europe, several countries (e.g., Norway, Ireland, Italy, Poland and Sweden) have not yet ratified laws for advance directives, whereas 15 countries have instated specific legislation. Regardless of legislation, the numbers of completed directives do not provide insights to the process of systematically improving communication skills and providing repeated ACP discussions. The implementation strategy, implementation outcomes and the definition of successful implementation are rarely described systematically in ACP studies [9, 43, 45].

### Facilitators and barriers

An important facilitator in the current study pertained to the communication and education about ACP as a crucial element of best clinical practice in NHs, which facilitated implementation and reduced the workload for the ambassadors. This is in line with facilitators identified by Livingston et al., who highlighted education and motivation as key facilitators [46]. Another crucial facilitator was the clear standards for the institutional structure, routines and staff responsibilities advocated in the COSMOS ACP intervention. This ensured that implementation was not dependent on one individual, but was anchored at the organizational level. This facilitator included several interesting subthemes, amongst which involvement of NH managers and unit leaders was crucial. We recruited NHs by motivating the top managers, who allocated resources to conduct the implementation adequately. Unlike the study by Sankaran et al. (2010), who reported that staff had difficulties attending the education [29], the managers were motivated to send their employees to the COSMOS education programme. This facilitator is also in agreement with Livingston et al., who highlighted the importance of motivated managers [46]. This strategy also answered previous findings suggesting that unclear responsibility might be a barrier for providing ACP [45].

Lack of time was identified as a main barrier in our study and has also been underlined as a major barrier in several studies on ACP in NHs [29, 45–47]. Although this is a common barrier, there is no clear answer as to how this challenge should be resolved. Alternative approaches such as focus on multidisciplinary staff competence, better organization of the NH services, and attractive working conditions for health-care students may be important contributions [48, 49]. Conflicting culture and opinions also represented an important barrier. Not all staff members were initially convinced that it was a good idea to include people with dementia in ACP discussion. The patients in our study had a low MMSE score, which complicates the implementation of ACP due to patients’ inability to communicate sufficiently and provide consent. At the same time, this is the reality in the today’s NH population, where most are multimorbid and have dementia, resulting in a high need for education in delivering ACP to people with reduced capacity [8, 22, 44, 48, 50, 51]. Importantly, the COSMOS education programme met this need, which subsequently influenced the feasibility, fidelity, and sustainability of the implementation [52, 53]. Some previous ACP intervention studies have included people with dementia [46, 54], others have not clearly specified cognitive status [47, 55], or only recruited cognitively intact residents [17, 43]. Dening et al. (2011) points out that ACP has benefits for people with dementia, a point which was clearly communicated in the COSMOS ACP education [22]. Another barrier pertained to lack of competence, of which lack of language comprehension was a challenge. Staff members of different nationalities demonstrated disparities in Norwegian language skills, which reduced the staff’s capability to understand and formulate sensitive questions. In general, unskilled staff showed lower motivation to engage in the implementation of ACP. Previous studies suggest that reluctant personnel represent a major barrier for implementing ACP [56, 57].

We argue that implementation of ACP is a necessity in today’s NH population. However, this study also has some limitations. The ACP intervention was multifold and time-consuming, which could preclude feasibility. Importantly, however, the required NH home staff attending the 2-day education seminar was achieved. The train-the-trainer approach did not have a multidisciplinary approach, as it was implemented as a part of the nurse’s daily routines. In addition, the inclusion rate of physicians was rather low for the 2-day education seminar. We do not know how many training sessions each unit managed to implement; however, as the researchers kept phone contact with the COSMOS ambassadors every second week, they were able to motivate the ambassadors when needed.

The implementation rate after only 4 months suggests that a sustained focus on ACP over time may ensure its implementation. The physicians’ involvement should ideally have been higher in our study, as they are responsible for the medical decision-making. ACP in the frail NH patients is complex, thus the communication need to be customized with respect for each individual [26]. This demands training of nurses and physicians to make the interdisciplinary team conscious of the complexity of ACP. Future studies may benefit from making a more top-down strategy for involving the NH physicians. Due to shift schedules and limited time, we could not assess all staff in each unit. Instead, we assessed the staff who participated in the data collection. This may have biased the data towards describing a more educated staff than generally present in the units.

This study has a comprehensive size and a variety of units, which promote generalizability. In addition, the present study provides detailed descriptions of the implementation process, the definition of successful implementation and information on the involvement of staff, which facilitates replicability and meaningful comparisons with future studies as well as the adaptability of the knowledge to the clinical field [9, 58].

## Conclusion

The content of ACP was considered relevant for the daily work in the unit, and the education seminars and material were often experienced as spot-on for practical use. Monthly communication with the family was the most frequently conducted communication from the patient log, and the predefined criteria for successfully implemented ACP were largely achieved. Nursing home routines and engagement of leaders and staff were crucial facilitators, whereas lack of time and competence limited the success of implementation.

## Additional files

Additional file 1: Traffic light used at the midway seminar. (PDF 49 kb)

Additional file 2: Advance Care Planning flash card front and back page. (PDF 327 kb)

## Abbreviations

ACP: Advance Care Planning
COSMOS: COmmunication, Systematic assessment and treatment of pain, Medication review, Organization of activities and Safety
MMSE: Mini-Mental Status Examination
NH: Nursing home
SD: standard deviation
