# Supplementary material for: Description of an advance care planning intervention in nursing homes: outcomes of the process evaluation
Source: BMC Geriatr. 2018 Jan 25;18:26. doi: 10.1186/s12877-018-0713-7 (PMC5785831; doi:10.1186/s12877-018-0713-7)
Supplement: Supplementary file 1 — Traffic light used at the midway seminar. (PDF 49 kb) [file 12877_2018_713_MOESM1_ESM.pdf]

Traffic light evaluation of ACP	
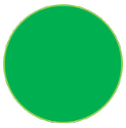	We have achieved a high level of competence and do not need help
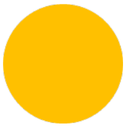	We have achieved some level of competence, but need to learn more
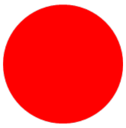	We have not achieved enough competence and for us this is not clear
