# Supplementary material for: Description of an advance care planning intervention in nursing homes: outcomes of the process evaluation
Source: BMC Geriatr. 2018 Jan 25;18:26. doi: 10.1186/s12877-018-0713-7 (PMC5785831; doi:10.1186/s12877-018-0713-7)
Supplement: Supplementary file 2 — Advance Care Planning flash card front and back page. (PDF 327 kb) [file 12877_2018_713_MOESM2_ESM.pdf]

ADVANCE CARE PLANNING

This is a repeating process where the patient together with relatives gets the opportunity to:

- Understand the disease and potential treatment alternatives
- Talk about his/her values, beliefs and earlier choices
- Identify his/her needs and goals for the stay
- Nominate a representative (surrogate) when the patient is no longer able to make decisions

WHEN AND HOW OFTEN?

- Patient, relatives, physician and care staff should have a conversation 2-3 weeks after the admission
- Have phone contact with relatives 1 time per month
- Regular follow up meetings every 3rd month
- Take active contact if changes in the disease, medical and treatment related questions and with hospital admission
- Be available when expressed needs for conversations from family and/or patient

WHO SHOULD PATICIPATE?

Patient, relatives, physician and caregivers should be included in advance care planning. Patient who explicitly do not want to take part, unconscious and persons with seriously behavioral disturbances should not participate.

HOW? 7 good questions for conversations

1. How involved have you been in the patients' treatment, care and decision making as family, and how much would you like to be included?
2. What have both of you (patient and family) understood about the situation and the disease?
3. What kind of additional information do both of you (patient and family) need to better understand the situation?
4. What should we know about the patient's life and values to ensure the best care? What matters and what makes life in general meaningful?
5. What goals, ideas and expectations do you both (patient and family) have for the nursing home stay?
6. Does the patient struggle with unfinished business?
7. Have both of you (patient and family) previously discussed end-of- life treatment e.g. hospitalization in case of acute illness?

TIPS FOR THE CONVERSATION

- Ask open ended questions and supplementary questions to understand the message:
  "What do you think about what we've talked about today?"
- When the patient cannot give informed consent, relatives should contribute to presumed consent, *"What would he/she have wanted in such a situation?"*
- Document the content of the conversation
- Carry out the measures that you agreed on

➡ The main goal is to build trust, confidence and contribute to predictability for all involved!

Read more in the guidelines!
